# An evaluation method for HMI of deep-sea manned submersible based on human reliability

**DOI:** 10.1038/s41598-023-41063-y

**Published:** 2023-09-04

**Authors:** Yao Zhou, Dengkai Chen, Jianghao Xiao, Hanyu Wang

**Affiliations:** Northwestern Polytechnical University, No. 127 West Youyi Road, Beilin District, Xi’an, 710072 Shaanxi Province China

**Keywords:** Electrical and electronic engineering, Mechanical engineering

## Abstract

Improving the human reliability of the human–machine interface (HMI) of deep-sea manned submersible is of great importance for the development of the deep-sea field. Based on the SHEL (Software S, Hardware H, Environment E, Liveware L) model, this study classifies the performance shaping factors (PSF) that affect the human reliability of submersible HMIs and builds a PSF system. The interpretative structural model (ISM) is used to matrix the interactions between the elements that make up the system of PSF. A multi-level recursive structure is obtained by building the corresponding adjacency matrix. The Noisy-OR model is introduced to construct a Bayesian network in order to build a new HMI evaluation method. A real case of Bayesian network causal inference verifies the validity of the built method. This study proposes a set of HMI human reliability evaluation methods applicable to deep-sea manned submersible, which provides a new idea for human reliability assessment.

## Introduction

The interior of a deep-sea manned submersible is confined and narrow. The special operating environment requires the submariner to judge and control the mission with experience and feedback from the equipment in a harsh and complex space. The HMI in the submersible mainly consists of the interface display and the console operation, which is the main medium of interaction between the submariner and the machine. Studies have shown that the main cause of HMI accidents is human factors^1^. The confined space, complex structure and high operational precision required inside the submersible make it extremely prone to human factors errors. Scholars have investigated the human reliability of HMI in many ways, such as Evica's analysis of the HMI in the cockpit of an aircraft using a systematic human factor error prediction method, which showed that low human reliability of the interface was the main cause of human errors in pilots^2^. Zhang et al. provided quantitative assessment data for the interface human reliability analysis of ship navigator by building an improved CREAM (Cognitive Reliability and Error Aanalysis Method, CREAM) model^3^. Zhang et al. optimises the design of the navigation interface of a deep-sea manned submersible to improve its interface usability from a human factor perspective^4^. Zhang et al. assessed the human reliability of submariners by establishing the probability of cognitive errors as a function of efficiency in a thermal environment^5^. Miao et al. decomposes the interactive interface of the manned submersible and optimally designs a new interface system from the perspective of optimal operational process^6^.

Methods of studies on human reliability are equally numerous, for example, Yuan et al. proposed a new controller interface-oriented human reliability analysis method based on the Delfino method and Bayesian networks, and the reliability of the method was verified by combining with actual cases^7^. Zhang et al. identified human reliability assessment models for the HMI by improving the CREAM method between controllers and pilots and between dispatchers and pilots, respectively^8, 9^. Hao et al. established a pilot human error analysis model from qualitative and quantitative perspectives based on the human reliability design requirements of the HMI in the aircraft cockpit, combined with the basic theory of cognitive behavior^10^. Wang et al. enhanced aircraft cockpit HMI human reliability by constructing a CPC (Common Performance Condition) effect-based fuzzy set and extending CREAM to calculate pilot cognitive failure probability^11^. Zhu et al. used fuzzy deduction and BP neural network and forward–backward algorithms to implement the reliability calculation of the HMI in the cockpit landing phase of a civilian aircraft^12^. The essential behavioral formation factors (PSF, Performance Shaping Factors) that affect human reliability have also been investigated. Kim et al. pointed out that the study of PSF in different contexts could significantly improve the human reliability of HMI in nuclear power plants^13^. Liu et al. redefined four types of PSF for nuclear power plant control rooms based on the expert correction method and successfully reduced the probability of human errors^14^. Yeong et al. used the CREAM analysis method to analyse HMI PSF in nuclear power plants, and the results showed that optimal HMI design and adequate training helped to improve operator performance^15^. Liu et al. established a basis for quantitatively studying the causal relationships between PSF by improving the Standardised nuclear Power plant Risk Analysis-Human reliability analysis (SPRA-H) method^16^. Yang et al. constructed a Bayesian network to predict controllers' probability of human error in multiple tasks using air control behavior formation factors as root nodes, and the results showed that Bayesian networks are more advantageous in studying this problem^17^. From the above, most scholars have analysed the essential PSF affecting human reliability, but few have studied the interactions between PSF. Bandeira pointed out that correlations between PSF are prevalent in complex civil air transport systems and that they have a significant impact on pilot performance and the success or failure of tasks related to flight procedures^18^. Obviously, the exploration of correlations between PSF is also one of the keys to improving the human reliability of HMI, but few studies have been carried out on the reliability of HMI for deep-sea submersible. Previous studies have only compared the sensitivities of different types of PSF, which not only lack comparability between the data, but also the conclusions obtained were not convincing. In the traditional study of PSF, only a single dimension is considered to affect human factor reliability. Most studies on human factor reliability were conducted in the dimension of "human" or "machine"^16^. Compared to analysing PSF from the perspective of individual factors, the use of different dimensional analyses allows for a better identification of the influential interactions between the various factors.

This study investigates the interactions between the factors that affect the HMI PSF of a deep-sea manned submersible. A more comprehensive and systematic evaluation method is built to improve the HMI human reliability of manned submersibles. It provides a more scientific and effective guidance for the design of the HMI while improving the operational efficiency of deep-sea manned submersible.

## Method

### HMI PSF for submersibles

The cockpit of an aircraft and a deep-sea submersible are both confined and complex human–machine environments. In 1972, Edward first proposed the principle of a specific system interface for "human" in safety work, which consists of the following elements: Software, Hardware, Environment and Liveware^19^. The initials of these four elements are used to represent the SHEL model. Errors tend to occur at the central point of contact between human and hardware, software, environment and liveware. The model depicts the vulnerability of modern production and is a direct guide to safety work. The interfaces described are not only found on the front line, but at all levels of the production organization, so the model is universally relevant. Based on the definition of the SHEL model, this study divided the elements covered by the submersible HMI into four aspects: system staff (L), system software (S), system hardware (H) and system environment (E), and the assessment was determined as a study of the interaction between *L–L, L–S, L–H* and *L–E*. A summary of the navigation-related literature and an interview survey with experts in the field of navigation yielded a total of 28 PSF, as shown in Table 1.

1. *L–L*: Study of the interactions between submariner and team members in terms of information exchange and operational collaboration capabilities.
2. *L–H:* Study of the interaction between submariner and hardware operational equipment.
3. *L–S:* Study of the interactions between submariner and software interfaces.
4. *L–E:* Study of the interactions between submariner and the operating environment of the submersible's working chamber.

**Table 1:** Deep-sea manned submersible HMI PSF summary.

Dimension	PSF	Description	References
*L–L*	*S*_*1*_*:* Physical performance	Individual’s physical performance is different, and good physical performance can help individuals better complete tasks. Such as vision, physical coordination, etc	^20–25^
	*S*_*2*_*:* Fatigue level	Fatigue is a common factor for operators, and long time operation will cause fatigue. Fatigue has been proved by many scholars to be a significant factor affecting work efficiency	
	*S*_*3*_*:* Emotional stat-us	Individual psychological and emotional performance. Emotions can be expressed through an individual's work state. Positive emotions are associated with high productivity, while negative emotions often lead to dangerous accidents	
	*S*_*4*_*:* Knowledge skills and performance	An individual's level of knowledge is a factor in determining job performance, and a high level of knowledge is usually associated with good performance	
	*S*_*5*_*:* Concentrate level	In the process of performing a task, the level of concentration is directly related to the successful operation of the task	
	*S*_*6*_*:* Awareness of work responsibility	The awareness of work responsibility refers to the individual's responsible attitude towards work. Individuals with strong awareness of responsibility will be aware of the coming danger and solve it in time to avoid accidents	
	*S*_*7*_*:* Reasonable st-aff selection and deployment	Reasonableness of the selection of submariner members and the deployment of positions in the team. Individual ability to meet the job requirements	
	*S*_*8*_*:* Clear division of labour and responsibility	Whether team members are clear about their roles and responsibilities. Many scholars have found that this factor hasan important impact on the completion of tasks	
	*S*_*9*_*:* Level of teamwork	The level of information exchange between team members and the level of operational cooperation, etc. Frequent exchange of information between members will reduce the occurrence of accidents	
*L–H*	*S*_*10*_*:* Information conveyed through digital interfaces	Digital interface is the main way of information transfer. Prominence display of important information on the digital interface, quick access to information, clarity, legibility and reliability of text and symbols, etc	^26–29^
	*S*_*11*_*:* Signs for directions	Accuracy and distinguishability of indicators and symbols in the display panel. These will affect the individual's capture of information, resulting in human error	
	*S*_*12*_*:* Display and control device layout	The visibility of the display, the accessibility of the control areas, the logical layout of the combi-nation of display and control areas, the functionality of the centre console adapted to the experience and habits of the submariner, etc	
	*S*_*13*_*:* General layo-ut of the space	Structural size of the working area, access and mobility space, etc. The narrow space of manned submersible will bring inconvenience to the operator, and the space layout plays an important role in reducing human error	
	*S*_*14*_*:* Seats & chairs	The suitability of the seat structure to the seating position and the comfort of the human spine. Sitting for long periods of time can cause discomfort in areas such as the waist and spin which can affect work performance	
	*S*_*15*_*:* Communication equipment	Adequacy of the working condition of the communication equipment, stability and clarity of the communication signals. The communication equipment inside the submersible is crucial, and the timely transmission of information to the corresponding posts is the key to preventing accidents	
	*S*_*16*_*:* Workstation alarm equipment	The clarity and meaning of the warning signals in the work cabin. When the warning message is obvious and easy to understand, the operator can quickly deal with the danger and avoid accidents	
	*S*_*17*_*:* Level of systematization and automation	The higher the level of system automation, the less the load on the staff, which can improve the situational awareness of the staff. The level of system automation is conducive to reducing human error	
*L–S*	*S*_*18*_*:* Integrity of t-he interface displ-ay	Information is displayed on the interface, whether all the key information needed by the staff can be displayed. A integrity display of information is available to support staff in making correct decisions	^7, 23,24,30,31^
	*S*_*19*_*:* Reasonablen-ess of the software feedback system	Whether the feedback from the software to the submariner is effective in motivating them to work. Reasonable software feedback system can improve the enthusiasm of staff, so as to work more actively	
	*S*_*20*_*:* Adequacy of software system training	Whether the work training and practical operation status covers all work scenarios. All software system functions should be fully trained for staff to reduce accidents caused by emergencies	
	*S*_*21*_*:* Integrity of t-he software opera-ting procedures	Whether the submersible submariner's procedures and specifications for performing operational tasks are adequate. A good software system should have detailed instructions for each step, so that the operator can complete the operation quickly	
	*S*_*22*_*:* Reasonablen-ess of system operation time	The running time of the system should conform to the rest habits of the operators. Too long operation time will bring fatigue to the operators, which will cause the resistance of the operators and lead to the occurrence of accidents	
	*S*_*23*_*:* Emergencies and preparedness	The integrity of the emergency response system and the reliability of the software implementation for responding to emergencies. The management of emergency situations is the key to preventing dangerous accidents	
	*S*_*24*_*:* System security level	The level of safety of the system is adequate for the psychological requirement of the submariner. Unsafe systems can lead to a psychological burden on operators when performing operations, which can lead to more human accidents	
	*S*_*25*_*:* System interconnection level	Whether the software system allows for interaction with other submariner members and collaboration in the accomplishment of tasks. The higher the level of system interconnection, the more frequent the communication between operators, thus reducing accidents	
*L–E*	*S*_*26*_*:* Microclimate	Whether the microclimate, such as air pressure, temperature, humidity and ventilation, is conducive to the physiological comfort of the submariner and improves operational efficiency	^29,30,32^
	*S*_*27*_*:* Lighting and color	Whether the lighting and colors are suitable for the visual recognition and communication of visual information to the submariner	
	*S*_*28*_*:* Noise and vibration	Whether noise and vibration are suitable for the hearing sensitivity, operational accuracy and emotional state of the submariner	

### A system of PSF for submersible HMI

A questionnaire was used to investigate and analyse the 28 PSF obtained to build a HMI human factor reliability PSF system for deep-sea manned submersible.

#### Questionnaire study

The questionnaire was administered to those who had experience in operating deep-sea submersibles (i.e. submariners, submarine trainees in training, etc.), were all male and had an average age of around 37 years old. The main information in the questionnaire consisted of basic information and PSF on human reliability, using a 5-point Likert scale, with 1 being "minimal impact" and 5 being "great impact". A small pre-sample survey was conducted to ensure the validity of the questionnaire before distribution. Before completing the questionnaire, we informed all participants of the purpose of the study and had them sign the questionnaire informed consent form. We prepared a small gift for each participant who completed the questionnaire. A total of 260 questionnaires were returned, of which 243 were valid, and a reliability check was conducted on the returned questionnaires to ensure the validity of the data. The demographic information from the questionnaire was shown in Table 2.

**Table 2:** Demographics of the questionnaire.

Question item	Options	Quantity	Proportion
Positions	Captain	12	4.6%
	Submariners	125	48.1%
	Submarine trainees	123	47.3%
Age(year)	18–25	96	36.2%
	26–30	109	41.8%
	31–40	50	19.2%
	41–50	15	5.8%
Education level	Postgraduate	154	59.2%
	Undergraduate	92	35.4%
	High school	12	4.6%
	Other	2	0.8%
Work experience(year)	< 1	96	36.9%
	1–3	129	49.6%
	3–5	24	9.3%
	5–10	7	2.7%
	10–20	4	1.5%

#### Usability testing of questionnaire

The reliability coefficient of the questionnaire as a whole was calculated by SPSS software to be 0.88, which indicates good consistency of the questionnaire. The same reliability test was conducted for the four pre-defined assessment dimensions in this study, and the results are shown in Table 3.

**Table 3:** Questionnaire reliability testing.

Interactive categories	Number of factors	*α* coefficient
*L–L*	9	0.94
*L–H*	8	0.86
*L–S*	8	0.82
*L–E*	3	0.95

The results in the table show that the alpha coefficients of the four assessment dimensions are *L–L *(0.94), *L–H *(0.86), *L–S *(0.82) and *L–E *(0.95), which were all greater than 0.6. According to the reliability test conditions of the questionnaire, the alpha coefficient is greater than 0.6, indicating that the factors present good consistency in all interactive categories and reach the requirements of the reliability test.

The 28 PSF were analysed for association validity with the four dimensions (*L–L, L–H, L–S, L–E*). The PSF for the four dimensions were *S*_*1*_*–S*_*9*_ (*L–L*), *S*_*10*_*–S*_*17*_ (*L–H*), *S*_*18*_–*S*_*25*_ (*L–S*) and *S*_26_*–S*_28_ (*L–E*), and the results of the analysis were shown in Table 4.

**Table 4:** Correlation analysis of PSF with dimensions.

PSF	Correlation analysis		PSF	Correlation analysis	
	R	Sig		R	Sig
*S*_*1*_	**0.492**	0.102	*S*_*15*_	0.745**	0.003
*S*_*2*_	0.636*	0.034	*S*_*16*_	0.853**	0.000
*S*_*3*_	0.904*	0.021	*S*_*17*_	0.764**	0.097
*S*_*4*_	0.797*	0.013	*S*_*18*_	0.886**	0.001
*S*_*5*_	0.623*	0.036	*S*_*19*_	0.744**	0.005
*S*_*6*_	0.865**	0.004	*S*_*20*_	0.622**	0.000
*S*_*7*_	0.394	0.069	*S*_*21*_	0.826**	0.004
*S*_*8*_	0.554	0.084	*S*_*22*_	0.867**	0.025
*S*_*9*_	0.846**	0.001	*S*_*23*_	0.775*	0.037
*S*_*10*_	0.768**	0.008	*S*_*24*_	0.743**	0.016
*S*_*11*_	0.819**	0.000	*S*_*25*_	0.632*	0.034
*S*_*12*_	0.267	0.002	*S*_*26*_	0.866*	0.014
*S*_*13*_	0.830**	0.009	*S*_*27*_	0.827*	0.027
*S*_*14*_	0.706**	0.004	*S*_*28*_	0.626**	0.000

**P < 0.01, *P < 0.05.

KMO (Kasier-Meyer-Olkin measure of Sample Adequacy) is the value of sampling appropriateness, which can determine the correlation and bias between sample data. The higher the KMO value, the stronger the correlation between the sample data. Bartlett's sphericity test can detect the independence relationship between variables. In this study, the questionnaire data obtained were analysed using SPSS software. The KMO test value for the questionnaire was 0.856 and the Bartlett's spherical test approximate chi-square was 1868.7. The data results obtained reached the requirements of the factor analysis. The initial component matrix was rotated using the maximum variance method to obtain the rotated component matrix. After removing the factors with factor loadings less than 0.6 (PSF number: *S*_*1,7,8,12*_) and multiple loadings greater than 0.2 from the rotation matrix, the data were retested for KMO values and Bartlett's spherical test.

#### PSF system

**Figure 1:**
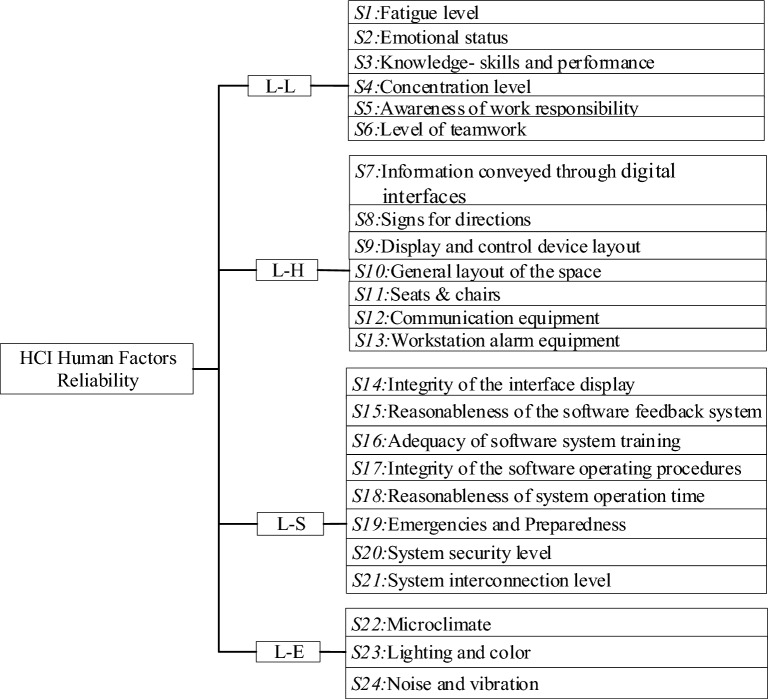
System of PSF for manned submersibles.

The four PSF that did not match the data test results were *Physical performance, Reasonable staff selection and deployment, Clear division of labour and responsibility, Display and control device layout.* After removing the unqualified data (Sig. *P* > *0.05*), all PSF were renumbered. A final system of PSF containing 4 dimensions was established. This system of indicators reflects the influence of the HMI of deep-sea manned submersibles on the behavioral operations of submariners, as shown in Fig. 1.

#### Ethical approvals

The study received ethical approval from the Human Research Ethics Committee of Northwestern Polytechnical University (Ref No: 245/2023). In addition, the Key Laboratory of Ergonomics of the Ministry of Industry and Information Technology of China and the Institute of Industrial Design of Northwestern Polytechnical University approved the use of the research site (Ref No: 24/2023). All relevant guidelines, procedures and regulations were followed. The experts involved in the study provided written informed consent. All participants were informed that they were free to withdraw from the study at any time without consequences.

## Model for human reliability evaluation

To identify the effects between the factors, this study combines an interpretative structural model with a Bayesian network to model the interactions of PSF for manned submersibles. Firstly, the interpreted structural model is used to obtain the hierarchical structure and map the model into a Bayesian network to complete the topology. Secondly, the Bayesian network data was populated by obtaining the prior probabilities of the root nodes and the conditional probabilities. Finally, a complete Bayesian network model was built to quantify the strength of the coupling interactions between the PSF.

### Interpretative structural models for PSF

**Figure 2:**
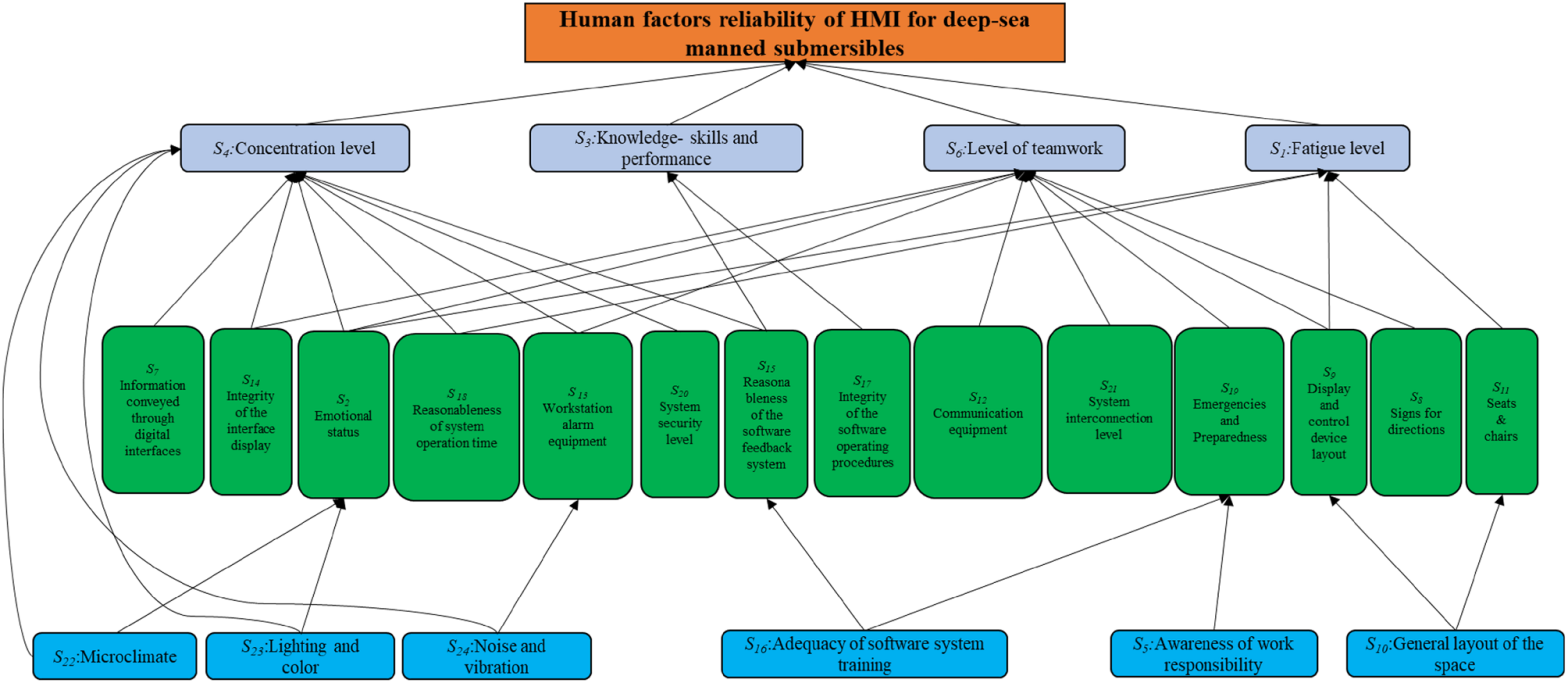
ISM of PSF.

ISM can build the correlation relationship between elements and achieve the building of multi-layer ladder models through matrix operations and directed graphs, and then obtain a clear system structure and hierarchy. In this study, we used ISM to sort out the PSF affecting human reliability, and determined the interactions between PSF factors by building reachability matrix. The classification of all PSF levels based on the reachability matrix. The relevant PSF factors were connected through directed arcs to build a ISM of PSF for the HMI of a deep-sea manned submersible, shown in Fig. 2.

As shown in Fig. 2, the ISM of PSF was divided into 3 levels. A hierarchical progressive interpretive relationship existed at each level from bottom to top. This study combined the four dimensions of *L–L, L–H, L–S* and *L–E* to analyse the model as follows:

1. The direct cause of errors were the first level. In other words, the submariner's fatigue level (*S*_*1*_), knowledge-skills and performance (*S*_*3*_), concentration level (*S*_*4*_), and level of teamwork (*S*_*6*_) in the *L–L* dimension were the direct causes of human-caused errors of the submariners.
2. The indirect causes of errors were the second level. In particular, the *L–L* dimension includes the factor of emotional status (*S*_*2*_). The *L–H* dimension includes the factors of information conveyed through digital interfaces (*S*_*7*_), signs for directions (*S*_*8*_), display and control device layout (*S*_*9*_), seats & chairs (*S*_*11*_), communication equipment (*S*_*12*_), workstation alarm equipment (*S*_*13*_). The *L–S* dimension includes the factors of integrity of the interface display (*S*_*14*_), reasonableness of the software feedback system (*S*_*15*_), integrity of the software operating procedures (*S*_*17*_), reasonableness of system operation time (*S*_*18*_), emergencies and Preparedness (*S*_*19*_), system security level (*S*_*20*_), system interconnection level (*S*_*21*_).
3. The deeper causes of errors were the third level. The awareness of work responsibility (*S*_*5*_) factor in the *L–L* dimension. The general layout of the space (*S*_*10*_) factor in the *L–H* dimension. The adequacy of software system training (*S*_*16*_) factor in the *L–S* dimension. The all factors (*S*_*22*_–*S*_*24*_) of *L–E* dimension.

### Human reliability analysis based on Bayesian networks

#### Fuzzification of node occurrence probabilities

**Figure 3:**
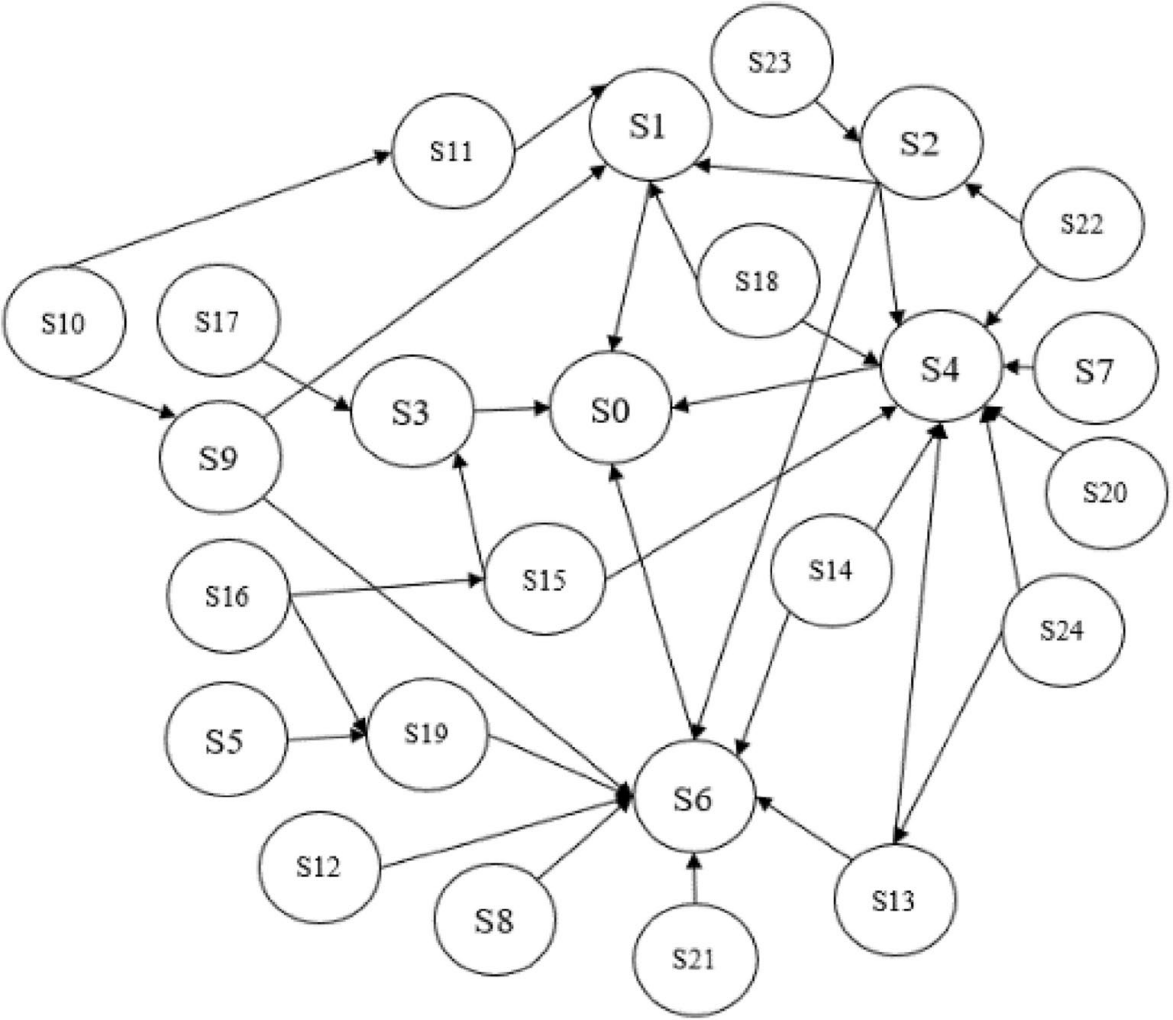
The ISM-based Bayesian network.

The model was adjusted using the causal graph correction method^33^. The final Bayesian network topology based on the interpreted structural model was established, as shown in Fig. 3.

This study assumed that each node in the network hierarchy consists of two states that have a positive and negative impact on human reliability. The node state settings and meanings were shown in Table 5. The mapping relationship between natural linguistic variables and fuzzy numbers was established using the natural linguistic variables description method, and the correspondence between linguistic variables and triangular fuzzy numbers is shown in Table 6.

**Table 5:** Meaning of all node states.

PSF	STATE = 0	STATE = 1	PSF	STATE = 0	STATE = 1
*S*_*1*_	Insignificant	Significant	*S*_*13*_	Good	Poor
*S*_*2*_	Positive	Negative	*S*_*14*_	Good	Poor
*S*_*3*_	Adequate	Inadequate	*S*_*15*_	Good	Poor
*S*_*4*_	Good	Poor	*S*_*16*_	Good	Poor
*S*_*5*_	Good	Poor	*S*_*17*_	Good	Poor
*S*_*6*_	Good	Poor	*S*_*18*_	Good	Poor
*S*_*7*_	Good	Poor	*S*_*19*_	Good	Poor
*S*_*8*_	Explicit	Ambiguous	*S*_*20*_	Good	Poor
*S*_*9*_	Effective	Effectiveness	*S*_*21*_	Good	Poor
*S*_*10*_	Good	Poor	*S*_*22*_	Adaptable	Inadaptable
*S*_*11*_	Comfortable	Uncomfortable	*S*_*23*_	Adaptable	Inadaptable
*S*_*12*_	Good	Poor	*S*_*24*_	Adaptable	Inadaptable

**Table 6:** Correspondence between natural language variables and triangular fuzzy numbers.

No	Semantic values	Triangular fuzzy number
1	VL (Very low)	(0,0,0.1)
2	L (Low)	(0,0.1,0.3)
3	ML (Medium low)	(0.1,0.3,0.5)
4	M (Medium)	(0.3,0.5,0.7)
5	MH (Medium high)	(0.5,0.7,0.9)
6	H (High)	(0.7,0.9,1.0)
7	VH (Very high)	(0.9,0.9,1.0)

#### Synthesis of fuzzy probabilities

When inviting experts to score, because each expert has a different educational background, knowledge base and level of perception, it can easily lead to conflicting opinions during the group's decision-making process. In this study, the Similarity Aggregation Method (SAM)^34^ was used to process the expert opinions in order to enable a consensus of expert opinions. The steps of SAM were as follows:

*Step 1*: Experts' similarity calculations for opinions.

Suppose the set of experts was ${E}_{k}(\mathrm{k}=\mathrm{1,2},\dots ,\mathrm{n})$, and ${R}_{u}$, ${R}_{v}$ were used to represent the opinions of any two experts, then ${\widetilde{R}}_{u}=\left({r}_{u1},{r}_{u2},{r}_{u3}\right)$ and ${\widetilde{R}}_{v}=({r}_{v1},{r}_{v2},{r}_{v3})$, and the similarity function ${S}_{uv}$ of experts ${E}_{u}$ and experts ${E}_{v}$ was shown in the formula (1). ${R}_{u}$ and ${R}_{v}$ were the standard triangular fuzzy numbers for expert opinion. The similarity function takes on a value between 0 and 1, with larger values representing higher similarity. In these formulas $\mathrm{k}$ is the number of experts. ${R}_{u}$ and ${R}_{v}$ represent the u and v experts, respectively. ${r}_{u1}$ represent the education level of the ${R}_{u}$ expert. ${r}_{u2}$ represent the knowledge level of the ${R}_{u}$ expert. ${r}_{u3}$ represent the perception level of the ${R}_{u}$ expert. ${r}_{v1}$ represent the education level of the ${R}_{v}$ expert. ${r}_{v2}$ represent the knowledge level of the ${R}_{v}$ expert. ${r}_{v3}$ represent the perception level of the ${R}_{v}$ expert.

1 $${S}_{uv}=1-\frac{1}{3}\sum_{i=1}^{3}\left|{r}_{ui}-{r}_{vi}\right|$$

*Step2*: Calculation of the average agreement of experts.

The average agreement ${A}_{A}({E}_{k})$ was calculated using the formula (2).

2 $${A}_{A}({E}_{k})=\frac{1}{k-1}{\sum }_{\begin{array}{c}v=1\\ u\ne v\end{array}}^{k}{S}_{uv}$$

*Step3*: Calculation of the relative agreement of experts.

The relative agreement ${R}_{A}({E}_{k})$ was calculated using the formula (3).

3 $${A}_{R}({E}_{k})=\frac{{A}_{A}({E}_{k})}{{\sum }_{k=1}^{n}{A}_{A}({E}_{k})}$$

*Step4*: The agreement factor for experts $C({E}_{k})$ was calculated as shown in formula (4).

In the formula: $w({E}_{k})$ being the weight of the expert; *β* is the slack factor, *β* = 0 when no expert weight is considered.

4 $$C({E}_{k})=\beta \cdot w({E}_{k})+(1-\beta )\cdot {A}_{R}({E}_{k})$$

*Step5*: The aggregation of expert opinion ${\widetilde{R}}_{AG}$ is calculated as shown in formula (5).

5 $${\widetilde{R}}_{AG}=\mathrm{C}({E}_{1})\cdot {\widetilde{R}}_{1}+\mathrm{C}({E}_{2})\cdot {\widetilde{R}}_{2}+\dots +\mathrm{C}({E}_{k})\cdot {\widetilde{R}}_{k}$$

The aggregation of PSF *S*_*14*_ was used as an example to illustrate the calculation process. Firstly, the semantic values of experts' fuzzy judgments for node *S*_*14*_ at "STATE = 1" were collected and then mathematically calculated according to the above method. After the aggregation of experts' opinions, the triangular fuzzy number of *S*_*14*_ was (0.00, 0.08, 0.21).

#### Defuzzification of data

Defuzzification is the mathematical calculation of fuzzy probabilities to obtain an exact value. In this study, the mean area method was used for defuzzification. The formula for defuzzification is shown in formula (6), where (*a, m, b*) represents a set of fuzzy numbers and *P* was the value after deconvoluting the triangular fuzzy numbers.

6 $$P=\frac{a+2m+b}{4}$$

The results for the root node *S*_*14*_:

7 $$P=\frac{0.00+2\times 0.08+0.21}{4}=0.09$$

#### Obtaining conditional probabilities for Bayesian networks

**Figure 4:**
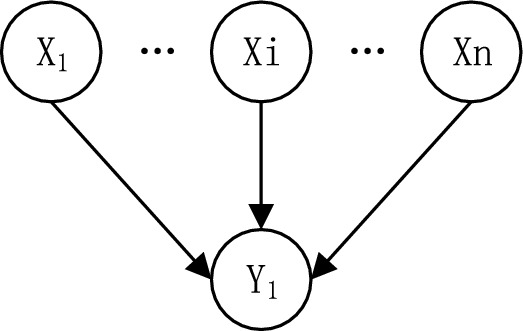
Diagram of the Noisy-OR model.

The Noisy-OR model can significantly reduce the number of parameters required to populate the CPT (Conditional Probability Table, CPT) of an event probability table in a Bayesian network. The Noisy-OR model was used in this study to describe the interaction between the cause of an event and its resulting impact. Suppose a binary variable *Y* which has *n* binary parents of *X*. Each variable has two states 0, 1 (0 for not occurring and 1 for occurring) as shown in Fig. 4.

After obtaining the conditional probabilities of child nodes independently influenced by parent nodes, the conditional probabilities of multiple parent nodes acting together can be calculated. The calculation formula was given in (8) and (9).

8 $$P(Y \leftarrow X_i)=\text P(\text B \textbar \overline{X}_1,\, \overline{X}_2...,\, {X}_i...,\, \overline{X}_n)$$

9 $$\text P(\text Y)=1 -\text P(\text y \leftarrow X_p)= \Pi_X {_i}\epsilon_X{_p}(1-P(y\leftarrow x_i))$$

In the formula: ${X}_{i}$ indicates that node ${X}_{i}$ occurs, $\overline{{X }_{i}}$ indicates that node does not occur; *X*_*p*_ indicates that simultaneous parent node union occurs; ${\text{P}}\left( {{\text{Y}} \leftarrow X_{i} } \right)$ indicates the probability of occurrence of node *Y* when parent node ${X}_{i}$ was independently influenced. The calculation process for node *S*_*4*_ was used as an example for illustration. The conditional probability that node S4 was under the influence of the parent node alone was:

*P*(*S*_4_ ← *S*_22_) = 0.34, P(*S*_4_ ← *S*_7_) = 0.27, P(*S*_4_ ← *S*_2_) = 0.58, P(*S*_4_ ← *S*_20_) = 0.65, P(*S*_4_ ← *S*_24_) = 0.77, P(*S*_4_ ← *S*_13_) = 0.46, P(*S*_4_ ← *S*_14_) = 0.75, P(*S*_4_ ← *S*_15_) = 0.35, P(*S*_4_ ← *S*_18_) = 0.52.

Calculate the conditional probability under the joint action of multiple parent nodes:

*P*(*S*_*4*_ ← *S*_*22*_, *S*_*7*_, *S*_*2*_, *S*_*20*_, *S24*, *S13*, *S*_*14*_, *S*_*15*_, *S*_*18*_) = 1 − [1 − *P*(*S*_*4*_ ← *S*_*22*_)]·[1 − *P*(*S*_*4*_ ← *S*_*7*_)]·[1 − *P*(*S*_*4*_ ← *S*_*2*_)]·[1 − *P*(*S*_*4*_ ← *S*_*20*_)]·[1 − *P*(*S*_*4*_ ← *S*_*24*_)]·[1 − *P*(*S*_*4*_ ← *S*_*13*_)]·[1 − *P*(*S*_*4*_ ← *S*_*14*_)]·[1 − *P*(*S*_*4*_ ← *S*_*15*_)]·[1 − *P*(*S*_*4*_ ← *S*_*18*_)] = 1 − (1–0.34)·(1–0.27)·(1–0.58)·(1–0.65)·(1–0.77)·(1–0.46)·(1–0.75)·(1–0.35)·(1–0.52) = 0.99.

### A case of human reliability analysis

A real-life case from the China Deep-sea Warrior manned submersible safety case compilation was selected for this study. According to the incident report, the submersible was on a 4500 m class sea trial. During the submersible's powered dive to sit on the bottom, the submariner failed to adjust the ballast water tank volume. The submersible's thrusters were underpowered triggering Inadequate Power's working chamber alarm, constituting a serious error event for the safety of a manned submersible.

#### Probability calculation of case events

**Figure 5:**
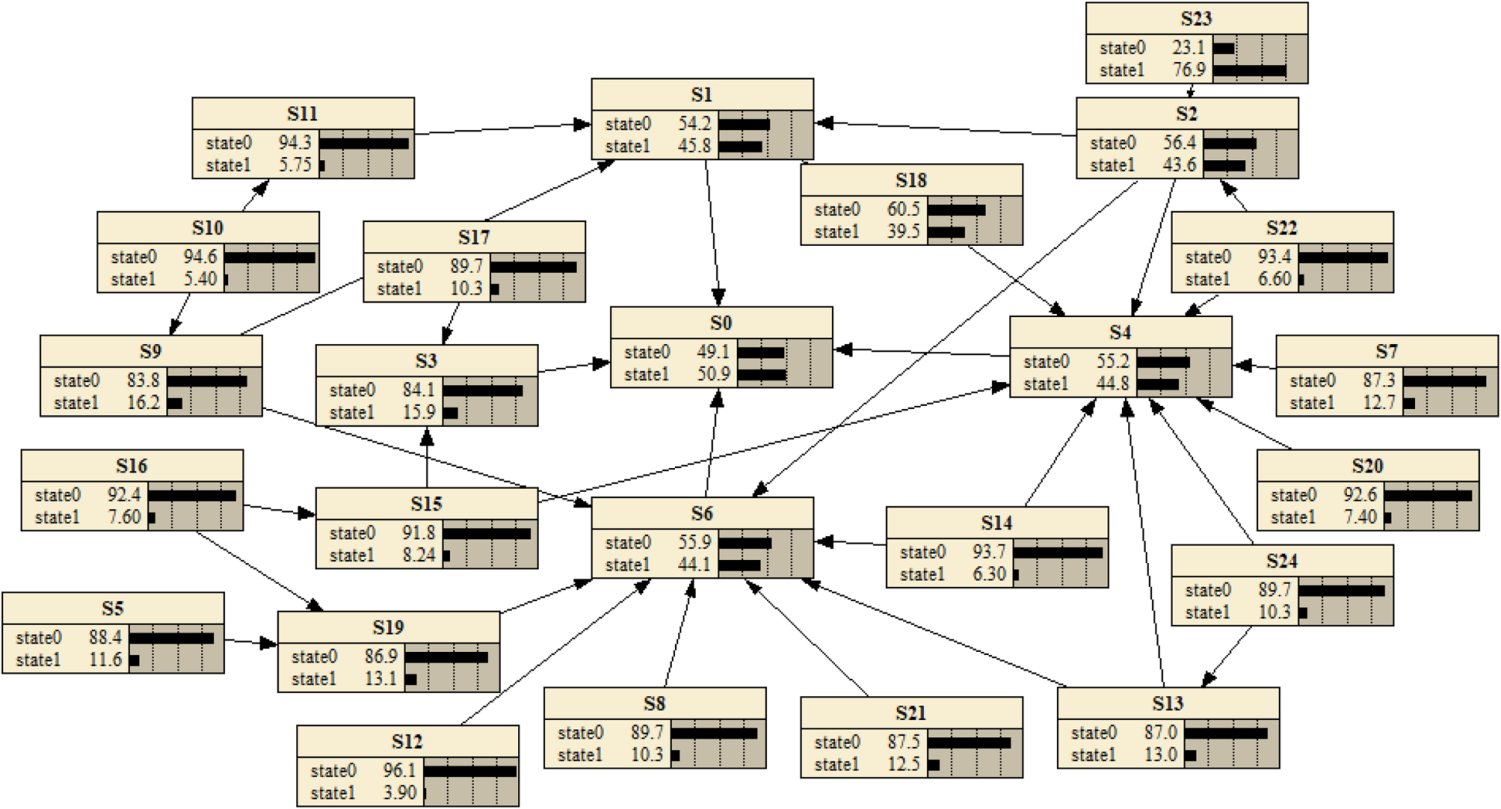
Diagram of Bayesian network causal inference. Data in a figure from *Netica V5.18* version of software to access the address: http://www.3h3.com/soft/163546.html.

Five experts in the field were invited to conduct interviews for this study. The experts gave fuzzy judgement values for PSF at "STATE = 1" based on practical experience and basic event information, and we used the formula to calculate the conditional probabilities under the influence of different combinations of parent nodes. As shown in Fig. 5, the human factor reliability (*S*_0_) probability for the HMI of this manned submersible was calculated to be 49.1% using *Netica* software, which is generally consistent with the state of the submersible during operation. *Netica* is the most widely used Bayesian network analysis software in the world.

The manned submersible was diving to sit on the bottom when the divers neglected to adjust the amount of water in the ballast water tanks. After the alarm the submariner recognized the error and ballast water was fed into the tanks in time for the subsequent dive to proceed normally without making a major error. This coincides with the results of this study and verifies the applicability of the proposed Bayesian network approach based on an interpreted structural model for the human reliability evaluation of the HMI of deep-sea manned submersibles.

#### Analysis of key PSF affecting human reliability

Suppose that the human interface of the manned submersible was in a negative state due to low human reliability. Set the state P(*S*_0_ = 1) = 100% of node *S*_0_, update the probability parameters of the network and get the posterior probability of each node. By comparing the prior probability with the posterior probability, the sensitive factors affecting the human factor reliability can be identified based on the before and after change values. The results obtained were shown in Table 7.

**Table 7:** Comparison of probabilities of each node of Bayesian networks.

Nodal variables	Prior probability	Posterior probability	Percentage change
*S*_*1*_	0.461	0.614	15.3%
*S*_*2*_	0.442	0.514	7.2%
*S*_*3*_	0.164	0.215	5.1%
*S*_*4*_	0.453	0.554	10.1%
*S*_*5*_	0.121	0.166	4.5%
*S*_*6*_	0.064	0.098	3.4%
*S*_*7*_	0.132	0.163	3.1%
*S*_*8*_	0.113	0.169	5.6%
*S*_*9*_	0.165	0.222	5.7%
*S*_*10*_	0.051	0.072	2.1%
*S*_*11*_	0.441	0.565	12.4%
*S*_*12*_	0.042	0.076	3.4%
*S*_*13*_	0.133	0.186	5.3%
*S*_*14*_	0.062	0.086	2.4%
*S*_*15*_	0.084	0.102	1.8%
*S*_*16*_	0.084	0.122	3.8%
*S*_*17*_	0.102	0.146	4.4%
*S*_*18*_	0.402	0.569	15.7%
*S*_*19*_	0.131	0.177	4.6%
*S*_*20*_	0.074	0.136	6.2%
*S*_*21*_	0.132	0.175	4.3%
*S*_*22*_	0.075	0.088	1.3%
*S*_*23*_	0.124	0.150	2.6%
*S*_*24*_	0.773	0.817	4.4%

## Discussion

In this study, a system of PSF was proposed, consisting of four different dimensions, L–L, L–H, L–S and L–E. The following discussion was conducted in this study.

### The fatigue level factor had the highest impact in the L–L dimension

The results of this study showed that individual fatigue was a key factor affecting the human reliability of the manned submersible human–machine interface, which was the same as the results found in many previous studies. Many safety incidents occur as a direct result of individual fatigue^35–37^. The small and confined space inside a manned submersible can easily cause submariner fatigue. Studies have shown that when operators return to work after a period of temporary absence from the task, it significantly increases staff resourcefulness, so appropriate breaks can be used as a risk management measure^38^. All submariners were tested for fatigue prior to entering the submersible, but due to the long duration of the dive and the small confined working area fatigue can easily be generated. Managers need to monitor submariner fatigue in order to develop effective management measures to cope with the demands of the submariner's position.

### The seats & chairs factor had the highest impact in the L–H dimension

In the hardware environment of the HMI, the seat & chairs was a key factor in the human factor reliability. This differs from the results of other studies. This is probably due to the small space inside the submersible and the predominantly sideways working position of the submariners. Such a position is not common in daily work, and prolonged lying on one's side is more likely to cause discomfort than a sitting position^39^. As a result, a higher level of design is required of the designers. The designers have to take into account the working characteristics and habits of the submariners and adopt a more humane design to meet the special requirements of the submarine process.

### The reasonableness of system operation time factor had the highest impact in the L–S dimension

At present, most of the ICAO member states have regulations on the maximum flight time and the duration of a single work session for pilots^40^. However, for the manned submersible field, there is no standard work duration regulation, moreover, there is a lack of detailed work time limits and arrangements. The work of submariners requires alternating day and night, which is physically demanding. Previous studies have pointed out that alternating day and night shifts require full consideration of human adaptability, with night and day shifts needing to be at least 48 h apart when they cross over^41^. The results of this study could provide insights into the development of the submersible field.

### The noise and vibration factor had the highest impact in the L–E dimension

Noise and vibration have emerged as key causes of psychological and physiological effects on individuals in confined human–computer interaction spaces. This is consistent with the model results presented in this study. The sound pressure level of the noise source can be controlled by, for example, arranging some sound insulation and absorption materials, vibration isolation and vibration absorption structures in the bulkhead of the submersible.

### Limitations

There are a number of limitations to the results of this study that may affect the generalisability of the model. Firstly, the initial identification of 28 PSF does not fully describe all the factors influencing human reliability. The human–machine interface of a manned submersible is constructed in a complex manner, which includes many other influencing factors. Although we obtained some important influencing factors through literature and expert interviews, more PSF will be included in the future to ensure the accuracy of the model as the internal design of the manned submersible is continuously updated. Secondly, this study has fuzzed the experts' opinions, and although some of the subjective differences can be removed to a certain extent, there is still some subjectivity, and it is important to remove as much error as possible from subjective results in future studies.

## Conclusion

This study analyses the human reliability of the HMI of deep-sea manned submersibles. By analyzing the relationship between four dimensions of PSF, we proposed a human reliability evaluation method for the human–machine interface of deep-sea manned submersible. Our innovation mainly includes the following aspects:

1. Four dimensions were selected to evaluate the human factor reliability of deep-sea manned submersible.
2. In addition to the effects of individual PSF on human factor reliability, we also analyzed the correlation effects between PSF.
3. The fatigue level factor had the highest impact in the *L–L* dimension. The Seats & Chairs factor had the highest impact in the *L–H* dimension. The Reasonableness of system operation time factor had the highest impact in the *L–S* dimension. The Noise and vibration factor had the highest impact in the *L–E* dimension.

The method allows for a more scientific evaluation study of the HMI of manned submersibles.

## Data Availability

The datasets used and/or analyzed during the current study are available from the corresponding author upon reasonable request.

## Abbreviations

HMI: Human–machine interface
PSF: Performance shaping factors
ISM: Interpretative structural model
